# Saline Injections in Infantile Diarrhœa

**Published:** 1913-08-02

**Authors:** George Burford

**Affiliations:** 35 Queen Anne Street, Cavendish Square, W.


### THE EDITOR'S LETTER-BOX.

### Saline Injections in Infantile Diarrhœa.

*To the Editor of* The Hospital.

Sir,—In the year 1910 Dr. Robert-Simon of Paris gave in an English dress his own experiences and observations with the isotonic sea-water of Quinton. Independent testimony also filtered into this country; and the writer, with a round half-dozen of professional colleagues, made a pilgrimage to Paris to watch the results of radium treatment in the hands of Wickham and Degrais, and of isotonised sea-water in the clinic of Quinton. Radium does not enter into the subject-matter of this letter, but the results of marine injections in the Quinton clinic fully corroborated those so strikingly detailed by Robert-Simon.

We worked through the clinics with patience and assiduity, and we were convinced that here was a remedial measure of high working values and generalised applicability. Among the legion of cases of infantile gastro-enteritis some stood out in unforgettable relief. An infant in arms suffering from this malady was emaciated and livid, with no corneal reflex, respirations counted as four to the minute, and no detectable radial pulse. The child lay inert and insensitive across its mother's knees. A subcutaneous injection of marine fluid was ordered, and in an hour's time we saw the child again. A transformation had been effected—the baby was now conscious, with bright eyes, the shadow of death no longer on its face. It seemed a veritable resurrection; and this success in varying intensity of malady we saw repeated in cases too numerous to record.

The clinic in its variety dealt with all ages and both sexes; and the maladies most amenable to this treatment were those originating in the gastro-intestinal tract, or manifesting themselves on the skin. In the rank and file of cutaneous cases some especially stood out as notable. An elderly man came with psoriasis, and his clinical photograph at the beginning of the treatment had shown a dissemination of the scaly spots practically all over the body. When stripped for our inspection the residues appeared chiefly in the axillary folds and the inter-gluteal cleft. After some weeks' treatment the major portion of his skin was free. A girl with specific ulcers over the tibiæ, said to have suffered with such for six years, and to have been treated continuously during this time, chiefly at the St. Louis, had the ulcerated surfaces healed in six weeks under sea-water injections. Another girl with an old-time tubercular skin lesion which had broken down, leaving a raw exposed surface in the neck, had obtained effective cicatrisation quite early in the course of treatment. These were more or less striking examples from the bulk of cases we saw at the clinic; and the average impression left on our minds was that we had seen the operation of a new remedial measure in diseased conditions too definite and repeated to admit doubt of its validity.

A similar clinic to ensure, if possible, similar results, was thereafter established in London, and its first year's work was detailed in the report which was issued last year. In 1912 M. Quinton visited Egypt, and during a stay of two and a half months personally superintended the introduction of his method of procedure in various hospitals and dispensaries in conjunction with the authorities. Lord Kitchener and Mr. Graham, the Director of Public Hygiene, took a personal interest in the work. Five "Dispensaires Quinton" were established, the Municipal Council of Alexandria voting 5,000 francs for the last.

Now as to results. The French Consul at Alexandria reports that at the first "Dispensaire Marin" two hundred and sixty infants had been received for treatment during the first month; of these the majority were in a grave, and a certain number in a desperate, condition. The cases were followed up; the mortality list was six, three of these succumbing to broncho-pneumonia. Very favourable reports have also been received from others of the Dispensaires.

I have no intention here of criticising in detail the work of Professor Day with so-called Quinton plasma. But I may suggest that the fluid used was not prepared after Quinton's method. It is not distilled water that Quinton uses to isotonise his fluid, a precaution rendered doubly judicious from the work lately done in England on the toxic properties of ordinary distilled water injected subcutaneously. And Quinton, instead of adopting the easy method of sterilisation by heat, expressly disallows it on the ground that it imparts a degree of toxicity to the fluid. It is scarcely surprising that Quinton's results could not be repeated if Quinton's methods were not adopted.—I am, yours truly,

George Burford, M.B.

35 Queen Anne Street, Cavendish Square, W.,
July 22, 1913.

[If Dr. Burford had followed a little more carefully Professor Day's article, or our own exceedingly brief abstract of it, he would have realised that Professor Day actually used Quinton fluid—straight from the fountain head—for one series of cases, as well as sea-water prepared somewhat after Quinton's method for another series, and Ringer's solution for a third. This sufficiently answers the last paragraph of Dr. Burford's letter. The former paragraphs are quite consistent with our own remarks (The Hospital, July 19, 1913, p. 474) and with Professor Day's results as we retailed them. It is not denied that injections of saline fluid do great good to certain cases of infantile diarrhœa: admittedly they do. We were merely interested to note that an expert of real eminence, after a careful trial, has been unable to find any greater virtue in Quinton's particular fluid (for which, by the way, the name of "plasma" is objectionable) than in certain other forms of saline fluid costing a minute fraction of the sums demanded for the former. Meanwhile, we note with some slight interest that the upholders of Quinton fluid seem not to be in perfect agreement with one another. For whereas Dr. Burford sticks to the original none-other-is-genuine product, Dr. J. R. Day, one of his fellow-workers, is now using a modified sea-water "made in England," and procurable at a somewhat less extortionate price. Lastly, we may add that the rest of the medical profession is unable to corroborate the views of the "round half-dozen" who went to Paris as to the superiority of Quinton fluid over all other saline injections, though, as we stated a fortnight ago, Robert-Simon's writings have certainly brought home to many doctors in Britain the value of such injections more fully than they had previously realised.—Ed. The Hospital.]

